# A TLR–CXCL1 pathway in DRG neurons induces neutrophil accumulation in the DRG and mechanical allodynia in EAE mice

**DOI:** 10.1038/s41598-019-48558-7

**Published:** 2019-08-19

**Authors:** Jing Zhang, Yuka Harada, Yoshinori Hayashi

**Affiliations:** 1 Department of Aging Science and Pharmacology, Faculty of Dental Science, Kyushu University, Fukuoka, Japan; 2 Department of Physiology, Nihon University School of Dentistry, Tokyo, Japan

**Keywords:** Neuroimmunology, Multiple sclerosis

## Abstract

Multiple sclerosis (MS) is a potentially disabling disease of the central nervous system. Approximately half of the patients with MS experience severe pain; however, currently available therapeutics provide only insufficient relief. The mechanisms underlying the generation of neuropathic pain in patients with MS are not fully understood. Recently, we found that neutrophil elastase from accumulated neutrophils in the dorsal root ganglion (DRG) sensitizes DRG neurons and induces mechanical allodynia in a mouse model of experimental autoimmune encephalomyelitis (EAE). However, the mechanism underlying neutrophil accumulation in the DRG after myelin oligodendrocyte glycoprotein (MOG_35–55_, immunogenic peptide) immunization remains unclear. Here, we found that C-X-C motif ligand 1 (CXCL1) was upregulated in DRG neurons after MOG_35–55_ immunization. Increased expression of CXCL1 protein was also observed in primary cultured DRG neurons treated with MOG_35–55_, which was mediated through toll-like receptor 4 (TLR4). Gene silencing of TLR4 or CXCL1 in DRG neurons significantly attenuated neutrophil accumulation in the DRG and mechanical allodynia during the preclinical phase of EAE (around day 5 after immunization). Our results thus suggest that a TLR4–CXCL1 pathway in DRG neurons triggers neutrophil recruitment in the DRG and subsequent mechanical allodynia in response to MOG_35–55_.

## Introduction

Multiple sclerosis (MS) is a progressive inflammatory disease that manifests as neurological deficits including motor, cognitive, and neuropsychiatric symptoms. Besides these symptoms, approximately half of the patients with MS experience severe pain including ongoing dysesthetic pain and paroxysmal pain^1,2^. However, current therapeutics, including antidepressants, anticonvulsants, and cannabinoid drugs, provide only insufficient relief of pain^2^. Development of new analgesics for MS-related pain can, thus, improve the patient’s quality of life.

Although there are no established pain models for ongoing dysesthetic pain and paroxysmal pain, mechanical allodynia and thermal hypersensitivity have been observed in experimental autoimmune encephalomyelitis (EAE, an animal model for MS), which is elicited by myelin oligodendrocyte glycoprotein (MOG_35–55_) immunization. Accumulated evidence indicates that mechanical allodynia is observed prior to the manifestation of motor dysfunction in EAE (from 9–12 days after immunization)^3–6^. In fact, neuropathic pain in patients with MS can appear prior to or immediately at the onset of neurological symptoms^7^. It is suggested that T cells and microglia play key roles in the development of neuropathic pain, because they are activated in the spinal dorsal horn (SDH) during the clinical phase of EAE^3,5^. The pro-inflammatory cytokines such as interleukin (IL)-1β, IL-6, and tumor necrosis factor α from non-neuronal cells facilitate neuronal excitability in the SDH^8^. However, it has been observed that mechanical allodynia caused by MOG_35–55_ immunization starts prior to the activation of non-neuronal cells in the SDH^3,5,6^. We previously found that the activation of neutrophils is synchronized with the induction of mechanical allodynia in MOG_35–55_-immunized mice. These cells accumulate in the dorsal root ganglion (DRG), which is a cluster of neurons in a posterior root of a spinal nerve that carries sensory information, but not in the central nervous system (CNS) including the spinal cord and brainstem. Furthermore, neutrophils synthesize neutrophil elastase (NE) in a cathepsin E-dependent manner, and NE sensitizes DRG neurons. Mechanical allodynia is completely abrogated in neutrophil-depleted mice during the preclinical phase of EAE (5 days after MOG_35–55_ immunization)^6^. However, the underlying mechanisms of neutrophil accumulation in the DRG during the preclinical phase of EAE remain unclear.

Chemokines, a superfamily of small pro-inflammatory proteins, trigger recruitment of leukocytes to the inflamed or damaged site. The chemokine (C-X-C motif) ligand 1 (CXCL1) is one factor contributing to the recruitment of neutrophils^9^, which is mediated by CXCR2^10^. A recent study found that the serum levels of CXCL1 are upregulated during the preclinical phase of EAE^11^. However, a direct link between the rise in CXCL1, neutrophil accumulation in the DRG, and mechanical allodynia during the preclinical phase of EAE has not been clarified.

The aim of this study was to elucidate whether an increment in CXCL1 protein levels in the DRG contributes to neutrophil accumulation and induces mechanical allodynia in MOG_35–55_-immunized mice.

## Results

### CXCL1 is upregulated in mouse DRG neurons during the preclinical phase of EAE

**Figure 1:**
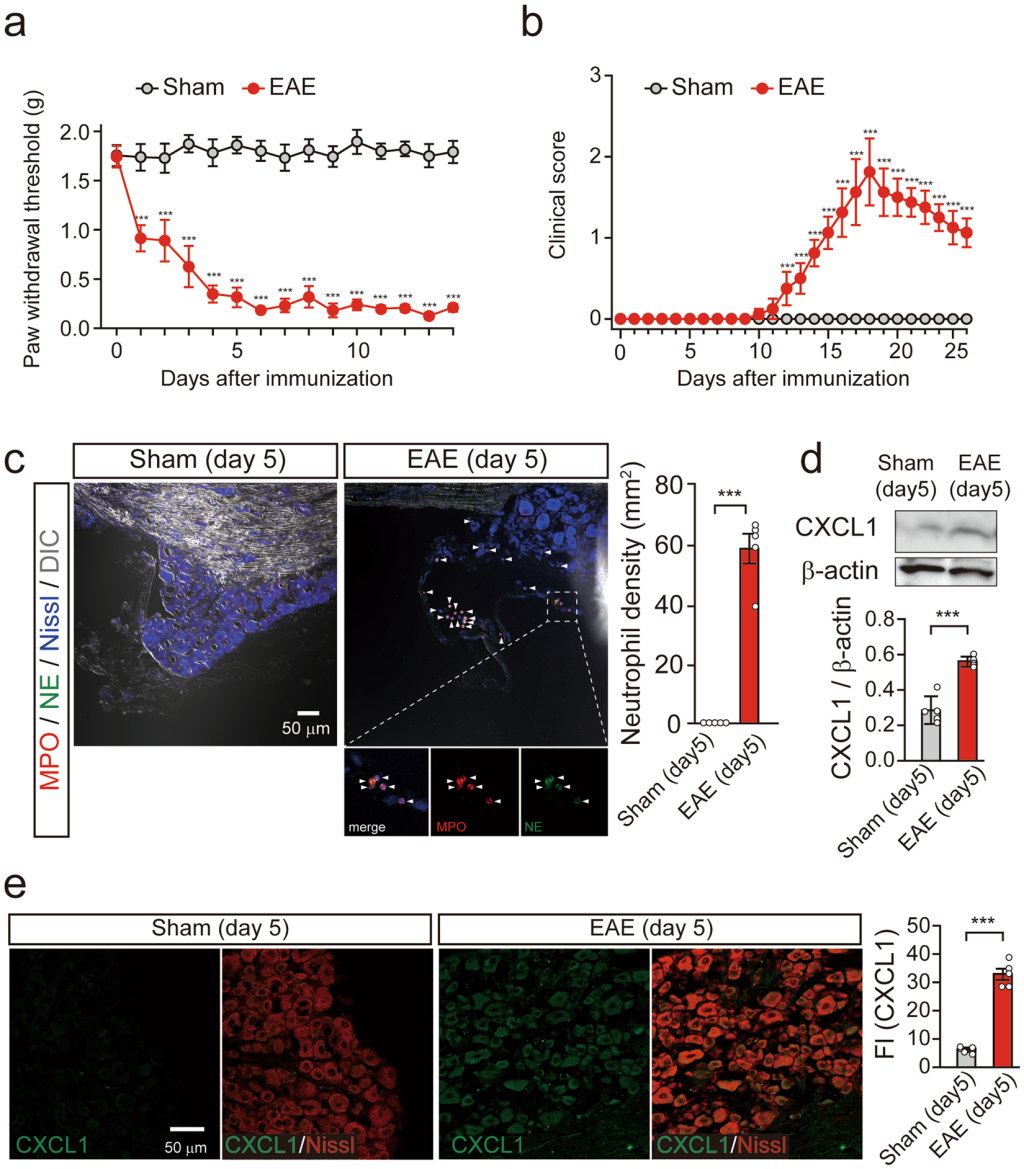
CXCL1 is upregulated in mouse DRG neurons after MOG_35–55_ immunization. (**a**,**b**) Time course of mechanical allodynia (**a**) and clinical score (**b**) in MOG_35–55_-immunized mice. n = 8 mice per group, two-way repeated measures ANOVA, F_(1, 210) MOG35–55 treatment_ = 1145, ***P < 0.001 in (**a**); n = 5 mice per group, two-way repeated measures ANOVA, F_(1,208) MOG35–55 treatment_ = 279, ***P < 0.001 in (**b**). (**c**) Neutrophil accumulation in the DRG 5 days after EAE induction in mice. Arrowheads indicate MPO (myeloperoxidase, red) and NE (neutrophil elastase, green) double-positive cells. The bottom images show enlarged images of the insets. DIC; differential interference contrast. Scale bar = 50 μm. Columns represent statistical data of neutrophil density in the DRG 5 days after MOG_35–55_ immunization. n = 5 mice per group, unpaired *t*-test, ***P < 0.001. (**d**) Immunoblot shows protein levels of CXCL1 in the DRG 5 days after MOG_35–55_ immunization. Columns represent statistical data of CXCL1 protein levels normalized to β-actin. n = 5 mice per group, unpaired *t*-test, ***P = 0.0003. (**e**) Fluorescent images of CXCL1 (gr**e**en) and Nissl (red) in the DRG 5 days after MOG_35–55_ immunization. Scale bar = 50 μm. Columns represent statistical data of fluorescence intensity (FI) of CXCL1 in the DRG neurons. n = 5 mice per group, unpaired *t*-test, ***P < 0.001. All values are the mean ± SEM.

To evaluate the role of CXCL1 in neutrophil accumulation in the DRG during the preclinical phase of EAE (<10–12 days after immunization), we analyzed whether CXCL1 protein was increased in the DRG of MOG_35–55_-immunized mice. Obvious mechanical allodynia, which was induced by von Frey filament applied to the hind paw, was observed at 4 days after MOG_35–55_ immunization (two-way repeated measures analysis of variance [ANOVA], F_(1,210) MOG35–55 treatment_ = 1145, ***P < 0.001; Fig. 1a). Motor disturbances were detected from 12 days after MOG_35–55_ immunization (two-way repeated measures ANOVA, F_(1,280) MOG35–55 treatment_ = 279, ***P < 0.001; Fig. 1b). These results show that mechanical allodynia during the preclinical phase of EAE preceded the motor disturbances. Neutrophil accumulation in the DRG of MOG_35–55_-immunized mice was further evaluated by immunohistochemical analyses using antibodies for MPO (myeloperoxidase) and NE, markers for activated neutrophils^12^. MPO/NE double-positive cells were detected in the DRG and its meninges at 5 days after MOG_35–55_ immunization (Fig. 1c), consistent with previous observations^6^. On the other hand, activated neutrophils were not observed in the DRG of non-immunized sham mice (Fig. 1c). We then analyzed the protein levels of CXCL1 in the lumbar 3–5 DRGs of MOG_35–55_-immunized or sham mice. The DRG collected from mice at 5 days after MOG_35–55_ immunization showed significantly higher levels of CXCL1 protein compared to that of sham mice (unpaired *t*-test, ***P < 0.001; Fig. 1d). In addition, immunohistochemical analysis found that intensity of CXCL1 immunofluorescence, which was merged with Nissl fluorescence (a marker for neurons), in the DRG slices was significantly increased at 5 days after MOG_35–55_ immunization (Fig. 1e). These results suggest that increased expression of CXCL1 in DRG neurons is synchronized with neutrophil accumulation in the DRG after MOG_35–55_ immunization.

### Neutrophils do not cause upregulation of CXCL1 protein in DRG neurons

**Figure 2:**
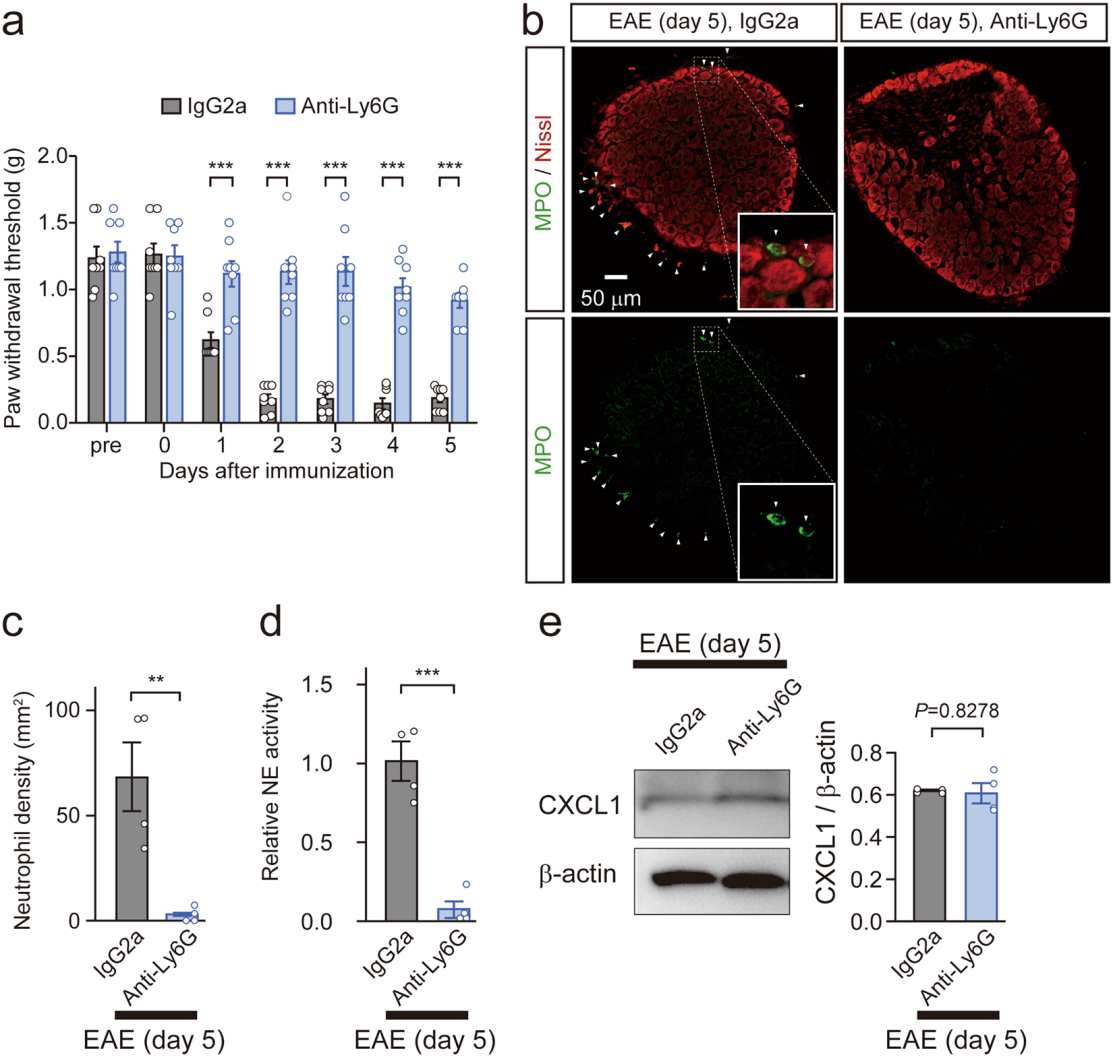
Induction of CXCL1 in the DRG is not dependent of neutrophil accumulation in the DRG. (**a**) Time course of mechanical allodynia in neutrophil-depleted mice after MOG_35–55_ immunization. Mice were treated with IgG2a (500 μg, intraperitoneally) or anti-Ly6G (500 μg, intraperitoneally) before MOG_35–55_ immunization. n = 8 mice per group, two-way repeated measures ANOVA, F_(1,98) antibody treatment_ = 217.8, ***P < 0.001. (**b**) Images show the immunofluorescence of MPO (green) and Nissl (red) in the lumbar 5 (L5) DRG of neutrophil-depleted mice on day 5 after MOG_35–55_ immunization. Arrowheads indicate MPO-positive cells. The inset indicates an enlarged image. Scale bar = 50 μm. (**c**) Columns represent statistical data of neutrophil density in the L5 DRG of neutrophil-depleted mice on day 5 after MOG_35–55_ immunization. n = 4 mice per group, unpaired *t*-test. **P = 0.0072. (**d**) Relative neutrophil elastase (NE) activity in the L5 DRG of neutrophil-depleted mice on day 5 after MOG_35–55_ immunization. n = 4 mice per group, unpaired *t*-test. ***P = 0.0003. (**e**) Immunoblot shows protein levels of CXCL1 in the DRG 5 days after MOG_35–55_ immunization in neutrophil-depleted mice. Columns represent statistical data of CXCL1 protein normalized to β-actin. n = 4 mice per group, unpaired *t*-test, n.s. *P* = 0.8278. n.s.: not significant. All values are the mean ± SEM.

We next asked whether the induction of CXCL1 in DRG neurons of MOG_35–55_-immunized mice was the result of neutrophil accumulation in the DRG. To address this possibility, we generated neutrophil-depleted mice using an intraperitoneal injection of anti-Ly6G mAb (clone 1A8, 500 μg), which did not influence the number of monocytes and lymphocytes^13,14^. Subsequently, the neutrophil-depleted mice were immunized with MOG_35–55_. Similar to previous observations^6^, neutrophil-depletion abrogated mechanical allodynia in MOG_35–55_-immunized mice (two-way repeated measures ANOVA, F_(1,98) antibody treatment_ = 217.8, ***P < 0.0001; Fig. 2a). MPO immunoreactivity in the DRG at 5 days after MOG_35–55_ immunization was not detected in anti-Ly6G mAb-treated mice (unpaired *t*-test, **P = 0.0072; Fig. 2b,c). Furthermore, the enzymatic activity for NE in the whole-cell lysates from the DRG of MOG_35–55_-immunized mice was abrogated by neutrophil depletion (unpaired *t*-test, ***P = 0.0003; Fig. 2d). Protein levels of CXCL1 in the DRG at 5 days after MOG_35–55_ immunization remained unchanged in the absence of neutrophils in the DRG (unpaired *t*-test, *P* = 0.8278 [not significant]; Fig. 2e), indicating that accumulated neutrophils in the DRG did not cause an increment in CXCL1 expression in DRG neurons. These results suggest that MOG_35–55_ directly stimulates DRG neurons and induces CXCL1 expression.

### MOG_35–55_ directly induces TLR4 in primary DRG neurons

**Figure 3:**
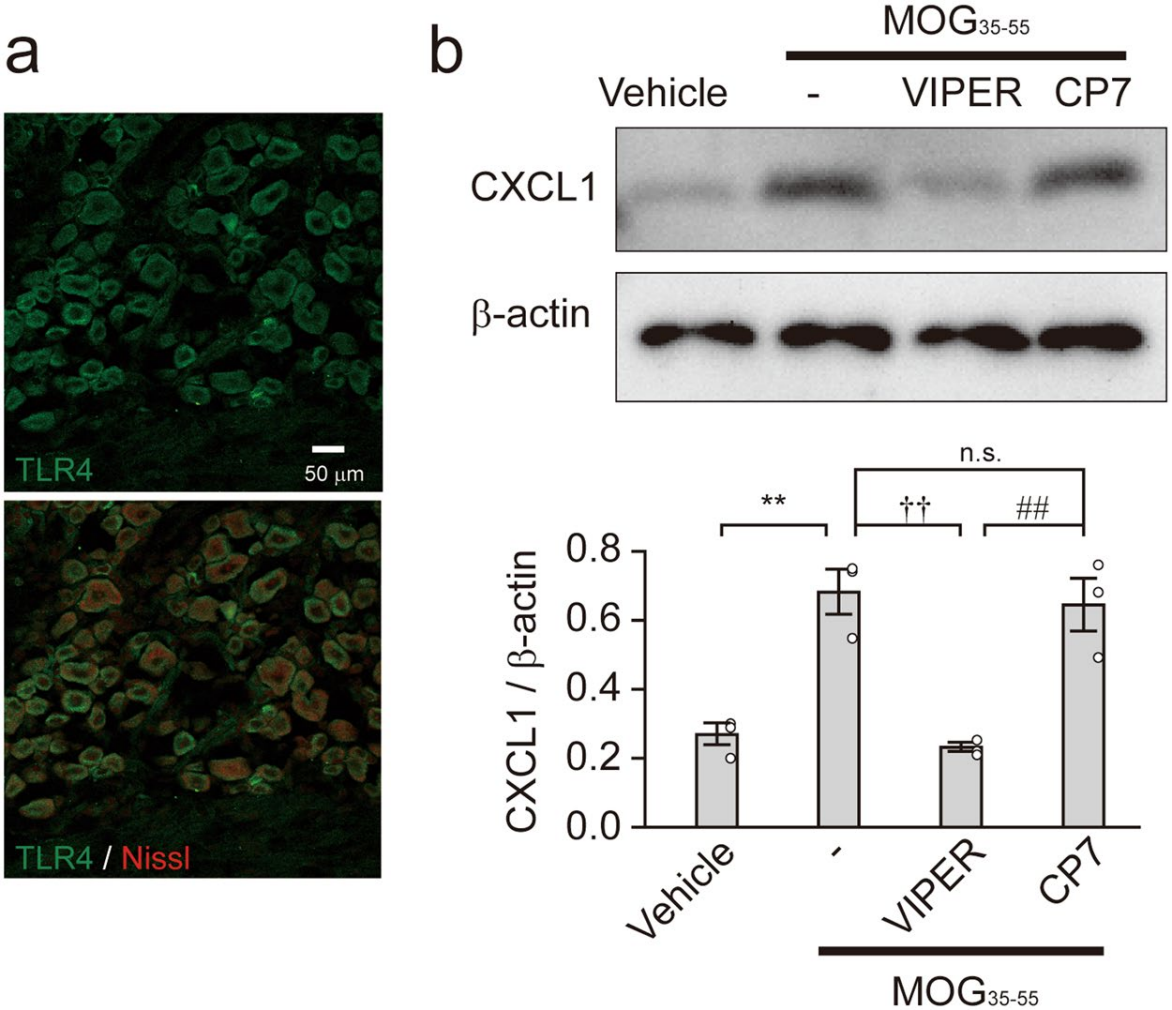
MOG_35–55_ induces CXCL1 through TLR4 in DRG neurons. (**a**) Immunofluorescence for TLR4 (green) and Nissl (red) in the DRG of naïve mice. Scale bar = 50 μm. (**b**) Immunoblot of CXCL1 in primary cultured DRG neurons. Primary cultured DRG neurons are treated with MOG_35–55_ (25 μg/mL) in the presence of VIPER (4 μM) or CP7 (4 μM). Columns represent statistical data of CXCL1 protein normalized to β-actin. n = 3 independent experiments. One-way ANOVA with Tukey’s test, Vehicle vs. MOG_35–55_: **P = 0.0027; MOG_35–55_ vs. MOG_35–55_ + VIPER: ^††^*P* = 0.0016; MOG_35–55_ + VIPER vs. MOG_35–55_ + CP7: ^##^*P* = 0.0028; MOG_35–55_ vs. MOG_35–55_ + CP7: n.s., *P* = 0.9543. n.s.: not significant. All values are the mean ± SEM.

We have previously identified a novel property of MOG_35–55_ (a CNS-derived peptide): it acts as a ligand for toll-like receptor 4 (TLR4)^6^. Given that a TLR4 pathway induces CXCL1 mRNA and protein^15,16^, we hypothesized that the induction of CXCL1 in DRG neurons of MOG_35–55_-immunized mice is mediated through TLR4 in DRG neurons. Therefore, we investigated the expression of TLR4 in DRG neurons of naïve mice by immunohistochemical analysis. TLR4 immunofluorescence in the DRG was merged with Nissl fluorescence (Fig. 3a), suggesting the existence of TLR4 in DRG neurons. This observation is consistent with the expression patterns of TLR4 in both rodent and human DRG neurons^17–19^. To analyze the direct interaction between TLR4 and CXCL1 in DRG neurons, we used primary cultured DRG neurons isolated from 3–4-week-old female mice, which were treated with MOG_35–55_ (25 μg/mL) for 6 h *in vitro*. MOG_35–55_ stimulation caused a 2.55-fold increase in CXCL1 protein in primary cultured DRG neurons (one-way ANOVA with Tukey’s test, vehicle vs. MOG_35–55_: **P = 0.0027; Fig. 3b). To investigate the involvement of TLR4 in the induction of CXCL1 in DRG neurons, we further treated primary cultured DRG neurons with VIPER (a specific inhibitor for TLR4, 4 μM) 1 h prior to MOG_35–55_ stimulation. VIPER significantly inhibited the increase in CXCL1 protein levels in primary cultured DRG neurons caused by MOG_35–55_ stimulation, whereas CP7 (a negative control for VIPER, 4 μM) did not (one-way ANOVA with Tukey’s test, MOG_35–55_ vs. MOG_35–55_ + VIPER: ^††^*P* = 0.0016; MOG_35–55_ + VIPER vs. MOG_35–55_ + CP7: ^##^*P* = 0.0028; MOG_35–55_ vs. MOG_35–55_ + CP7: *P* = 0.9543 [not significant]; Fig. 3b). These results suggest that induction of CXCL1 in DRG neurons after MOG_35–55_ immunization is mediated through TLR4.

### A TLR4–CXCL1 pathway in DRG neurons triggers neutrophil accumulation in the DRG of MOG_35–55_-immunized mice

**Figure 4:**
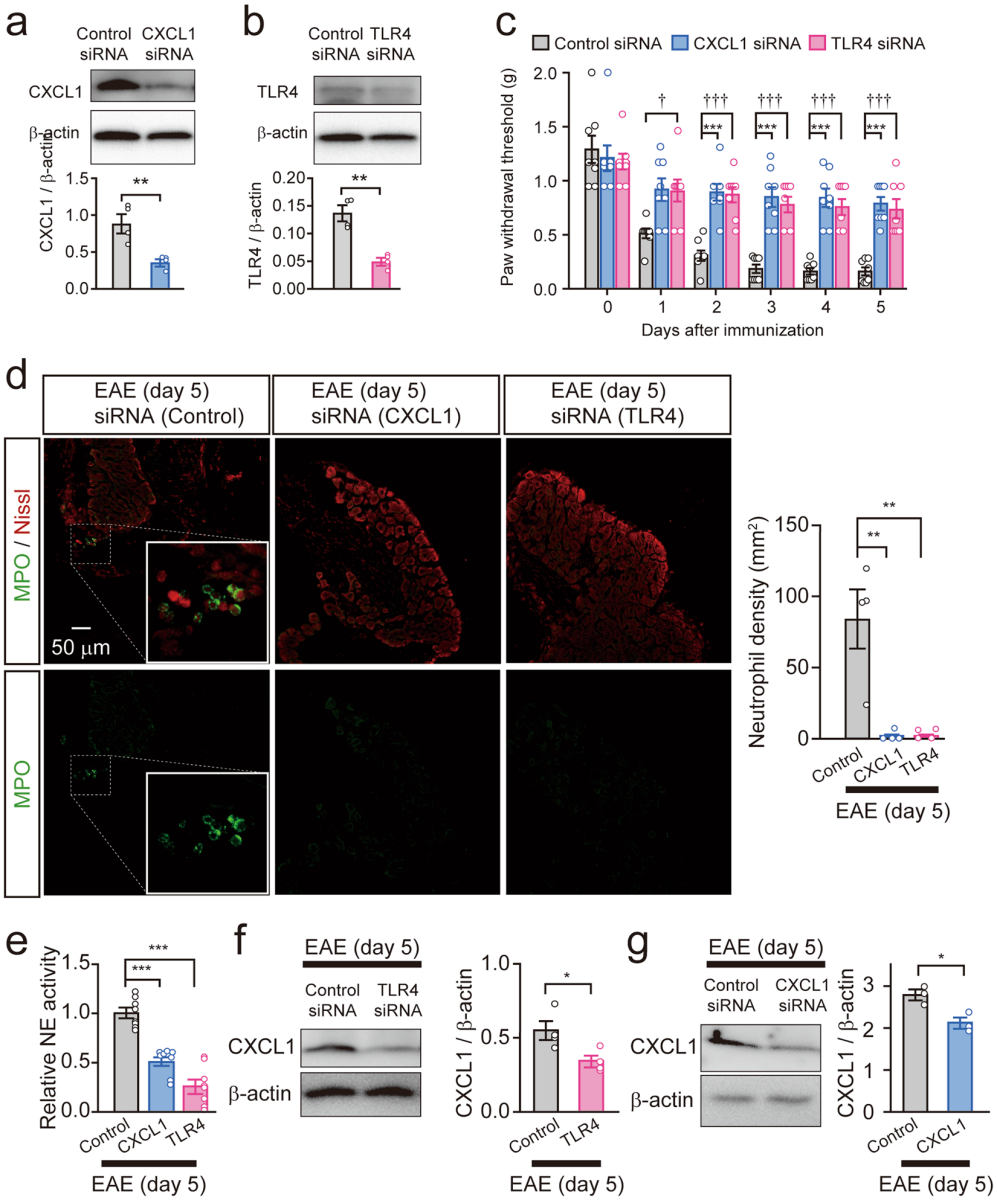
Gene silencing of CXCL1 or TLR4 in the DRG attenuates neutrophil accumulation in the DRG. (**a**,**b**) Knockdown efficacy of CXCL1 or TLR4 siRNA in the DRG collected from naïve mice 5 days after the first siRNA treatment. Immunoblot shows CXCL1 (**a**) and TLR4 (**b**) in the DRG of siRNA-treated mice. Columns represent statistical data of CXCL1 (**a**) and TLR4 (**b**) protein normalized to β-actin. n = 4 mice per group, unpaired *t*-test, **P = 0.0079 in (**a**), **P = 0.0012 in (**b**). (**c**) Time course of mechanical allodynia after MOG_35–55_ immunization in control and CXCL1- or TLR4-knockdown mice. n = 8 mice per group, two-way ANOVA with Tukey’s multiple comparisons test. Control siRNA vs. CXCL1 siRNA, day 1: *P* = 0.0535; day 2: ***P = 0.0002; days 3–5: ***P < 0.0001; Control siRNA vs. TLR4 siRNA, day 1: ^†^*P* = 0.0417; days 2–5: ^†††^*P* < 0.0001. (**d**) Images show the immunofluorescence of MPO (green) and Nissl (red) in the DRG 5 days after EAE induction in siRNA-treated mice. The inset indicates an enlarged image. Scale bar = 50 μm. Columns represent statistical data of neutrophil density in the DRG 5 days after MOG_35–55_ immunization. n = 4 mice per group, one-way ANOVA with Dunnett’s test; Control siRNA vs. CXCL1 siRNA: **P = 0.0018; Control siRNA vs. TLR4 siRNA: **P = 0.0020. (**e**) Relative neutrophil **e**lastase (NE) activity in the DRG 5 days after MOG_35–55_ immunization. n = 8 mice per group, one-way ANOVA with Dunnett’s test; Control siRNA vs. CXCL1 siRNA: ***P < 0.0001; Control siRNA vs. TLR4 siRNA: ***P < 0.0001. (**f**,**g**) Immunoblot shows protein levels of CXCL1 in the DRG 5 days after MOG_35–55_ immunization in TLR4- (**f**) or CXCL1-knockdown mice (**g**). Columns represent statistical data of CXCL1 protein normalized to β-actin. n = 3–4 mice per group, unpaired *t*-test, *P = 0.0318 (**f**) and *P = 0.0216 (**g**). All values are the mean ± SEM.

To assess whether a TLR4–CXCL1 pathway in DRG neurons contributes to neutrophil accumulation in the DRG during the preclinical phase of EAE, we performed *in vivo* knockdown of *Tlr4* or *Cxcl1* genes in DRG neurons. To eliminate the possible involvement of TLR4 or CXCL1 in immune cells^20,21^, we performed local knockdown of the target genes in the DRG using small interfering RNA (siRNA), and not knockout mice. The knockdown efficacy of siRNAs was examined by immunoblot analyses of the DRG collected from naïve mice subjected to intrathecal injection of Silencer Select siRNAs for 4 consecutive days. Intrathecal injection of corresponding siRNA exhibited reduction of CXCL1 (57.6 ± 7.4%) and TLR4 proteins (63.4 ± 6.3%) in the DRG compared to control siRNA treatment (unpaired *t*-test, Control siRNA vs. CXCL1 siRNA: **P = 0.0079; Control siRNA vs. TLR4 siRNA: **P = 0.0012; Fig. 4a and b). Using CXCL1- or TLR4-knockdown mice, we assessed mechanical allodynia during the preclinical phase of EAE. The siRNAs did not affect basal nociception (two-way ANOVA with Tukey’s multiple comparisons test, Control siRNA (day 0) vs. CXCL1 siRNA (day 0): *P* > 0.9999; Control siRNA (day 0) vs. TLR4 siRNA (day 0): *P* > 0.9999; Fig. 4c). Control siRNA-treated mice showed significant reduction in paw withdrawal threshold (PWT) after MOG_35–55_ immunization (two-way ANOVA with Tukey’s multiple comparisons test, day 0 vs. days 1–5: ***P < 0.0001; Fig. 4c). On the other hand, the reduction in PWT after MOG_35–55_ immunization was significantly attenuated in CXCL1- or TLR4-knockdown mice (two-way ANOVA with Tukey’s multiple comparisons test, Control siRNA vs. CXCL1 siRNA, day 1: *P* = 0.0535; day 2: ***P = 0.0002; days 3–5: ***P < 0.0001; Control siRNA vs. TLR4 siRNA, day 1: ^†^*P* = 0.0417; days 2–5: ^†††^*P* < 0.0001; Fig. 4c). We further analyzed the number of activated neutrophils in the DRG at 5 days after MOG_35–55_ immunization. The number of activated neutrophils in the DRG of CXCL1- or TLR4-knockdown mice was significantly lower than that of control siRNA-treated MOG_35–55_-immunized mice (one-way ANOVA with Dunnett’s test, Control siRNA vs. CXCL1 siRNA: **P = 0.0018; Control siRNA vs. TLR4 siRNA: **P = 0.0020; Fig. 4d). The reduced number of neutrophils in the DRG led to diminished NE activity. As expected, NE activity in the DRG of CXCL1- or TLR4-knockdown mice at 5 days after MOG_35–55_ immunization was significantly lower than that of control siRNA-treated MOG_35–55_-immunized mice (one-way ANOVA Dunnett’s test, Control siRNA vs. CXCL1 siRNA: ***P < 0.001; Control siRNA vs. TLR4 siRNA: ***P < 0.001; Fig. 4e). We finally tested whether increased expression of CXCL1 in the DRG after MOG_35–55_ immunization can be prevented in TLR4-knockdown mice. TLR4 siRNA as well as CXCL1 siRNA significantly inhibited the induction of CXCL1 in the DRG after MOG_35–55_ immunization (unpaired *t*-test, *P = 0.0318; Fig. 4f and unpaired *t*-test, *P = 0.0216; Fig. 4g, respectively). In addition, immunofluorescence of CXCL1 and TLR4 in the DRG at 5 days after EAE induction was attenuated by siRNA treatment (Supplementary Fig. 1). These results suggest that CXCL1 in DRG neurons triggers the recruitment of neutrophils through TLR4, which induces mechanical allodynia after MOG_35–55_ immunization.

## Discussion

In the current study, we have demonstrated that MOG_35–55_ immunization induces upregulation of CXCL1 protein in DRG neurons, which was also observed in neutrophil-depleted mice. We have previously identified MOG_35–55_ as a TLR4 ligand^6^. An increment in CXCL1 protein was mediated through TLR4 in primary cultured DRG neurons. Using an *in vivo* knockdown model, mechanical allodynia and neutrophil accumulation following MOG_35–55_ immunization were significantly attenuated via TLR4–CXCL1 signaling in DRG neurons. We have also previously demonstrated that accumulated neutrophils are able to activate DRG neurons by releasing NE, which generated nociceptive information^6^. This neuroimmune crosstalk led to the generation of mechanical allodynia during the preclinical phase of EAE.

It is largely accepted that T-helper 17 (Th17) cells are involved in various autoimmune diseases, including EAE^22^. IL-17A, which is mainly released from Th17 cells, is involved in nociception in the nerve-injured model^23^ and EAE model^4^. In addition, IL-17A is one factor contributing to the recruitment of neutrophils^24^. Therefore, IL-17A might contribute to neutrophil accumulation in the DRG during the preclinical phase of EAE. However, we did not detect T cells in either the DRG or SDH 5 days after MOG_35–55_ immunization^6^, consistent with the findings of Frezel *et al*.^5^. Immune cell infiltration in the CNS is restricted to the clinical phase of EAE^6^. Therefore, the recruitment of neutrophils in the DRG during the preclinical phase of EAE is not due to T cells. However, we could not exclude the involvement of circulating T cells in nociception after MOG_35–55_ immunization^4^.

Besides DRG neurons, tissue-resident macrophages and mast cells possibly induce CXCL1 and trigger neutrophil accumulation in the DRG. It is known that TLR4-mediated activation of macrophages and mast cells in the DRG can trigger nociception^25,26^. Furthermore, these cells are able to produce CXCL1 in response to lipopolysaccharide, a ligand for TLR4^10^. Despite the lack of direct evidence of the involvement in EAE-induced neuropathic pain, studies have implicated the participation of these cells in mechanical allodynia in EAE, as described below. Transient receptor potential melastatin 2 (TRPM2) is widely expressed in immune cells including monocytes, macrophages, neutrophils, and T cells^27–29^. Mechanical allodynia during the preclinical phase of EAE was found to be attenuated in TRPM2-deficient mice^30^. The activation of mast cells was identified in the meninges within 1 day after MOG_35–55_ immunization, which was observed prior to neutrophil recruitment^31^. From these observations, resident macrophages and mast cells in the DRG may be other factors underpinning the recruitment of neutrophils after MOG_35–55_ immunization.

CXCL1 and TLR4 are expressed not only in the DRG but also in glial cells in the spinal cord^32–34^. Therefore, intrathecally injected siRNA possibly influences CXCL1 and TLR4 in the spinal cord. CXCL1 is known to be involved in neuropathic pain after nerve injury^32^. Localization of CXCL1 in the SDH is restricted to astrocytes, and its upregulation depends on astrocyte activation^32^. On the other hand, the activation of astrocytes in the spinal cord was not observed during the preclinical phase of EAE (Supplementary Fig. 2). These evidences indicate that CXCL1 in astrocytes does not contribute to the generation of mechanical allodynia during the preclinical phase of EAE. TLR4 is expressed in microglia, which are believed to be a potent therapeutic target for neuropathic pain caused by nerve injury^34^. However, Sorge *et al*. demonstrated that a TLR4 pathway in the spinal cord is limited to pain models in male mice^33^. More recently, sex-specific difference in pain perception has been found to be attributed to different immune cells; hence, microglia-mediated signaling is not found in female mice^35^. Moreover, female mice were used for EAE experiments in the current study, since MS is three times more common in women than in men^36^. In addition, microglia in the spinal cord are not yet activated during the preclinical phase of EAE^6^. Considering the above observations, we excluded the possible involvement of CXCL1 and TLR4 in the spinal cord on neutrophil accumulation in the DRG, and mechanical allodynia during the preclinical phase of EAE.

Based on the data that neutrophil-depleted mice did not show mechanical allodynia, it is evident that neutrophils certainly play an important role in the generation of mechanical allodynia during the preclinical phase of EAE. Naïve mice never show mechanical allodynia under physiological condition, although neutrophils are located in the DRG^37^. Distinct from previous data, we could not observe MPO/NE immunoreactivity in the DRG of sham mice. This is due to the properties of antibodies. The anti-Gr-1 antibody, which recognizes membrane-surface antigens, can detect resting-state neutrophils^38^. On the other hand, MPO and NE are released from activated neutrophils^12^. Considering the involvement of NE on mechanical allodynia^6^, accumulation of activated neutrophils in the DRG, and not the total number of neutrophils, is more accurate to assess the role of neutrophils on mechanical allodynia during EAE. Given the partial attenuation of mechanical allodynia by CXCL1- or TLR4-knockdown in DRG neurons or inhibition of NE released from accumulated neutrophils in the DRG^6^, circulating neutrophils and accumulated neutrophils in the DRG additively contribute to the mechanical allodynia during the preclinical phase of EAE. Activated neutrophils cause disruption of the blood–brain barrier (BBB) and blood–spinal cord barrier (BSCB) in EAE mice. Immune cell infiltration in the CNS is a pathophysiological hallmark of patients with MS and EAE mice. Aubé *et al*. found that neutrophil depletion delays EAE onset and its severity and reduces BSCB permeability^39^. BSCB breakdown leads to infiltration of T cells and macrophages in the CNS^39^, which induce demyelination^40^. The significance of neutrophils in patients with MS has been suggested in clinical studies. An increased number of neutrophils has been observed in the serum of patients with MS, although granulocytes are rare in mature MS lesions^11,41^. In addition, an increased level of NE has been observed in the serum of patients with MS, which is due to enhanced degranulation of neutrophils^11,41^. NE is now known to cause increased vascular permeability in a mouse model of ischemia^42^. The increased level of NE in patients with MS possibly decreases the integrity of the BBB and BSCB. From the above observations, it can be acknowledged that pain therapy based on NE during the early phase of MS might reduce the severity of motor dysfunction by prevention of BBB and BSCB dysfunction.

Activation of DRG neurons through TLR4 is not restricted to EAE. Neuropathic pain is also associated with sickle cell disease, which is a group of disorders that affects hemoglobin in red blood cells. It is known that heme, a derivative of hemoglobin after hemolysis, can act as a TLR4 ligand^43^, and neutrophils have been shown to participate in neuropathic pain in sickle cell disease^44^. In addition, accumulation and TLR4-mediated activation of mast cells in the DRG have also been observed in this disease mice model^26^. Thus, a TLR4 pathway might trigger neuroimmune crosstalk in the DRG.

In conclusion, the current study suggests that MOG_35–55_ induces CXCL1 in DRG neurons via TLR4, with the net result being neutrophil recruitment and the generation of mechanical allodynia during the preclinical phase of EAE.

## Methods

### Animals

Female mice were used for all the experiments, since MS is most frequently diagnosed in women^36^. C57BL/6 mice (3–4 and 10–12 weeks old) were purchased from CLEA Japan (Tokyo, Japan). All animals were housed at a temperature of 22 ± 1 °C with a 12-h light–dark cycle (light on 8:00–20:00) under specific pathogen-free conditions and fed food and water *ad libitum*. All animal experiments in this study were approved by the Institutional Animal Care and Use Committee of Kyushu University (Protocol Numbers: #A26-12-0 and #A30-249-1). All methods were performed in accordance with the relevant guidelines and regulations. They were also in accordance with the ethical guidelines of the International Association for the Study of Pain^45^.

### Immunization

Mice were immunized with subcutaneous injection of 50 µL emulsion containing MOG_35–55_ (MEVGWYRSPFSRVVHLYRNGK, 300 µg, GenScript) and complete Freund’s adjuvant (CFA, 300 µg, Difco Laboratories) with heat-inactivated *Mycobacterium tuberculosis* H37Ra (300 µg, Becton Dickinson) in the bilateral inguinal region. Pertussis toxin (PTX, 500 ng, Sigma) was injected intraperitoneally at the time of immunization and 2 days after MOG_35–55_ immunization. For the negative control experiments, mice were immunized with CFA/PTX.

### Behavioral test

All mice were habituated to the testing environment for 3 days and were tested for mechanical allodynia. The room temperature remained stable at 22 ± 1 °C. Calibrated von Frey filaments (0.02–2.0 g; North Coast Medical, Inc.) were applied to the midplantar surface of the hind paw^6,46,47^. The 50% paw withdrawal thresholds (PWT) were calculated using the up-down method^48^. Each mouse was tested on both left and right hind paws and the average score was calculated. For measuring clinical scores, mice were monitored daily according to the severity, which was graded as follows: 0 = normal; 1 = paralyzed tail; 2 = loss of coordinated movement, hind limb paralysis; 3 = paralysis of both hind limbs; 4 = fore limb paralysis; and 5 = moribund. Investigators were blinded to the genotype of mice and treatment.

### Depletion of neutrophils in mice

Anti-Ly6G mAb (clone 1A8, 500 μg, BP0075-1, BioXcell) or isotype control rat IgG2a (clone 2A3, 500 μg, BP0089, BioXCell) were injected intraperitoneally into naïve C57BL/6 mice (8–12 weeks old) on days 0 and 3 after MOG_35–55_ immunization. Behavioral testing was started 1 day before initial injection of antibodies and conducted for 5 days after initial injection of antibodies.

### Primary cultured DRG neurons

C57BL/6 mice (3–4 weeks old) were deeply anesthetized with pentobarbital (200 mg/kg, intraperitoneally). Then, the lumbar 3–5 DRGs were collected. The DRGs were digested for 50 min in a 1 mg/mL collagenase Type II (Worthington Biochemical Corporation) in Hank’s Balanced Salt Solution at 37°C and following treatment with 0.05% of trypsin-EDTA solution (Thermo Fisher Scientific) for 15 min at 37°C. After trituration, the DRGs were suspended with DMEM/F12 (Thermo Fisher Scientific), which contained 50 ng/mL mouse β-nerve growth factor (NGF, BioLegend). DRG neurons were placed in a dish coated with poly-l-lysine (500 μg/mL, Sigma-Aldrich, Merck KGaA) and laminin (1 μg/mL, Corning). Following 2 days in culture, primary DRG neurons were treated with MOG_35–55_ (25 μg/mL) for 6 h. In some experiments, they were treated with VIPER (a specific inhibitor for TLR4 that interacts directly with the TLR4 adaptor proteins MyD88 adaptor-like and TRIF-related adaptor molecule, 4 μM, IMGENEX)^49^ or CP7 (a negative control peptide of VIPER, 4 μM, IMGENEX)^49^ 1 h prior to MOG_35–55_ stimulation. The effective concentration of MOG_35–55_ and VIPER was determined according to previous findings^6^.

### Western blot

The lumbar 3–5 DRGs and primary cultured DRG neurons were lysed in lysis buffer (10 mM Tris-HCl: pH 7.4, 150 mM NaCl, 1% Triton X-100, 0.5% NP-40, and protease inhibitor cocktail) and mixed with sample buffer. Proteins (20 μg) were loaded into each lane and separated by 10% sodium dodecyl sulfate polyacrylamide gel electrophoresis (SDS-PAGE) gel. After transfer, the membranes were blocked with TBS-T (0.2% Tween-20 in TBS) containing 5% Blocking One (Nakarai Tesque, Kyoto, Japan) for 1 h at room temperature, and then incubated with the primary antibodies diluted in TBS-T containing 5% Blocking One for overnight at 4 °C. The following primary antibodies were used: anti-myeloperoxidase antibody (1:2,000; cat. no. AF3667, R&D Systems), anti-mouse NE/ELA2 antibody (a marker for NE, 1:2,000; AF4517, R&D Systems), anti-CXCL1 antibody (1:2,000; cat. no. AF453, R&D Systems), anti-TLR4 antibody (1:2,000; cat. no. 19811-1-AP, Proteintech), and anti-β-actin antibody (1:10,000; cat. no. ab8226, Abcam). After being washed with TBS-T, the membranes were incubated with horseradish peroxidase-conjugated secondary antibody (1:1,000; GE Healthcare) for 2 h at room temperature. The membrane-bound horseradish peroxidase-labeled antibodies were detected using Immobilon ECL Ultra Western HRP Substrate (Merck Millipore) with an image analyzer (LAS-4000, Fuji Photo Film Co.). The bands that were analyzed by apparent molecular size were quantified using the ImageJ software program (http://rsbweb.nih.gov/ij/). The band intensity was normalized to β-actin.

### Immunohistochemistry

Immunohistochemistry was conducted according to the method described previously^6^. Briefly, 10-µm sections were cut from the lumbar 5 DRG, which was collected 5 days after MOG_35–55_ immunization. In some experiments, the lumbar 5 DRG was collected from CXCL1- or TLR4-knockdown mice 5 days after MOG_35–55_ immunization. Blocking was achieved using 1% normal donkey serum (Jackson ImmunoResearch), 1% bovine serum albumin (Sigma), and 0.1% Triton-X (Sigma) in phosphate-buffered solution (PBS) for 1 h. The specimens were incubated with anti-CXCL1 antibody (1:1,000), anti-MPO antibody (1:400), anti-mouse NE/ELA2 antibody (1:200), or anti-TLR4 antibody (1:200) overnight at 4 °C. The specimens were washed three times with PBS, and then stained with the secondary antibodies conjugated with Alexa 488 (1:400; Jackson ImmunoResearch) or Cy3 (1:400; Jackson ImmunoResearch), NeuroTrace™ 435/455 Blue Fluorescent Nissl Stain (1:1000; N21479, Thermo Fisher Scientific), or NeuroTrace™ 530/615 Red Fluorescent Nissl Stain (1:1000; N21482, Thermo Fisher Scientific) for 2 h at 4 °C. For the staining of the spinal cord, 40-µm lumbar 5 spinal cord slices were incubated with anti-glial fibrillary acidic protein (1:2,000; cat. no. Z0334, DAKO) for 3 days at 4 °C. After washing with PBS, the specimens were stained with Secondary antibody conjugated with Alexa 488 (1:400) for 2 h at 4 °C. The specimens were mounted in the anti-fading medium Vectashield (Vector Laboratories). Images were captured using by a C2si Confocal Laser Microscope (Nikon). A region of interest (ROI) was drawn around the Nissl-positive cells. Fluorescence intensity (FI) was measured by ImageJ software. The average of FI in each neuron was taken as the FI value of one image.

### Intrathecal injection

Under isoflurane anesthesia, a 30-gauge needle was inserted into the intrathecal space according to the method described previously^6,46,47^. The site of injection was the groove between L5 and L6 of the vertebral column. Control (Silencer™ Negative Control, 20 pmol/5 μL, Thermo Fisher Scientific), CXCL1 siRNA (20 pmol/5 μL, cat. no. s67078, Thermo Fisher Scientific), or TLR4 siRNA (20 pmol/5 μL, cat. no. s75207, Thermo Fisher Scientific) were mixed with Lipofectamine RNAiMAX (Thermo Fisher Scientific) and injected intrathecally once a day for 4 consecutive days. On the following day, the lumbar 3–5 DRGs isolated from siRNA-treated mice were collected and the knockdown efficacy of siRNAs was evaluated by western blot analysis. MOG_35–55_ immunization was started after 4 days of siRNA treatment. The lumbar 3–5 DRGs were collected 5 days after MOG_35–55_ immunization in CXCL1- or TLR4-knockdown mice and analyzed using western blot and immunohistochemistry according to the above-described methods.

### Measurement of elastase activity

Elastase activity in whole-cell lysates from lumbar 3–5 DRGs of neutrophil-depleted mice and siRNA-treated mice 5 days after MOG_35–55_ immunization was examined using the NE-specific synthetic substrate *N*-methoxysuccinyl-Ala-Ala-Pro-Val p-nitroanilide (600610, Cayman Chemical). Total concentration of protein in each specimen was determined by BCA assay. A 10-µg sample was incubated with 1 mM in 0.1 M Tris-HCl buffer (pH 8.0) containing 0.5 M NaCl and 1 mM substrate for 1 h at 37 °C. Then, p-nitroaniline absorbance was measured by a microplate reader (Infinite M200 Spectrophotometer, Tekan) at 405 nm. Relative NE activity was normalized to that of the IgG2a- or Control siRNA-treated group.

### Statistics

Reagents treatment, behavioral test, and statistical analyses were separately and blindly conducted. All data are shown as the mean ± standard error of the mean (SEM). Data normality was assessed using the Shapiro–Wilk test. The statistical analyses were performed using a one-way ANOVA followed by post *hoc* Dunnett’s test or Tukey’s test, two-way ANOVA with post *hoc* Tukey’s test, and unpaired *t*-test using the GraphPad Prism 7 software program. Detail statistical methods and *P* values are described in the Results section and the figure legends. Unless otherwise indicated, the data met the assumptions of equal variances. Differences were considered to be significant for values at *P* < 0.05.

## Supplementary information

Supplementary Figure

## Data Availability

All data generated or analyzed during this study are included in this published article and its Supplementary Information Files.
