# Supplementary material for: A TLR–CXCL1 pathway in DRG neurons induces neutrophil accumulation in the DRG and mechanical allodynia in EAE mice
Source: Sci Rep. 2019 Aug 19;9:12003. doi: 10.1038/s41598-019-48558-7 (PMC6700073; doi:10.1038/s41598-019-48558-7)
Supplement: Supplementary file 1 — Supplementary Figure [file 41598_2019_48558_MOESM1_ESM.pdf]

A TLR4-CXCL1 pathway in DRG neurons induces neutrophil accumulation in the DRG and mechanical allodynia in EAE mice

Jing Zhang[1], Yuka Harada[1], and Yoshinori Hayashi[1,2]

[1]Department of Aging Science and Pharmacology, Faculty of Dental Science, Kyushu University Fukuoka, Japan

[2]Department of Physiology, Nihon University School of Dentistry, 1-8-13 Kandasurugadai, Chiyoda-ku, Tokyo, Japan

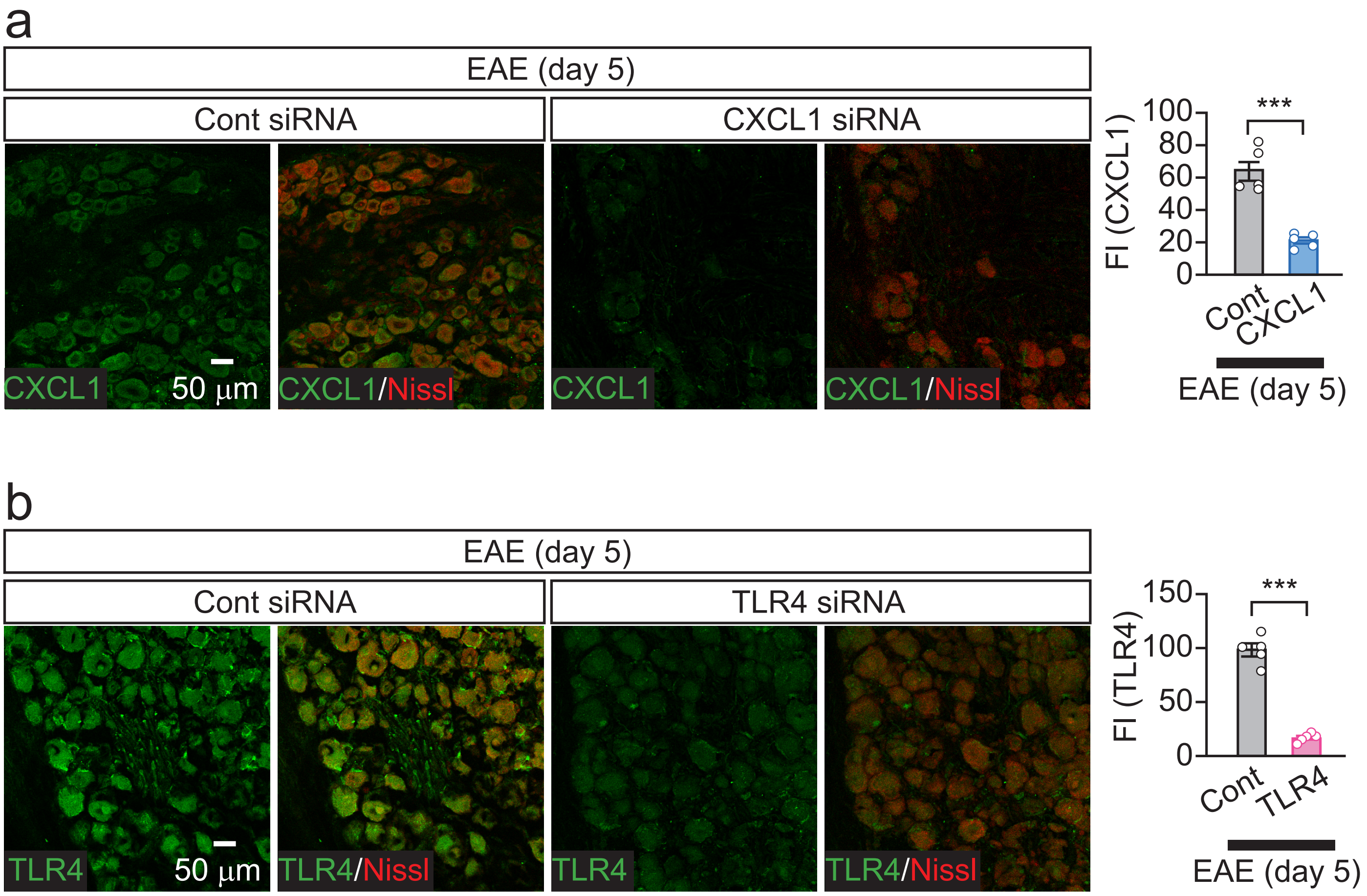

Supplementary Figure 1. Fluorescent images for CXCL1 (**a**) and TLR4 (**b**) in the DRG 5 days after $MOG_{35\text{-}55}$ immunization in CXCL1- or TLR4-knockdown mice. Columns represent statistical data of fluorescence intensity (FI) of CXCL1 (a) and TLR4 (**b**) in DRG neurons. Green fluorescence indicates CXCL1 or TLR4. Red fluorescence indicates Nissl. n = 5 mice per group, unpaired *t*-test, $***P = 0.001$ (a) and $***P < 0.001$ (**b**). All values are mean ± SEM.

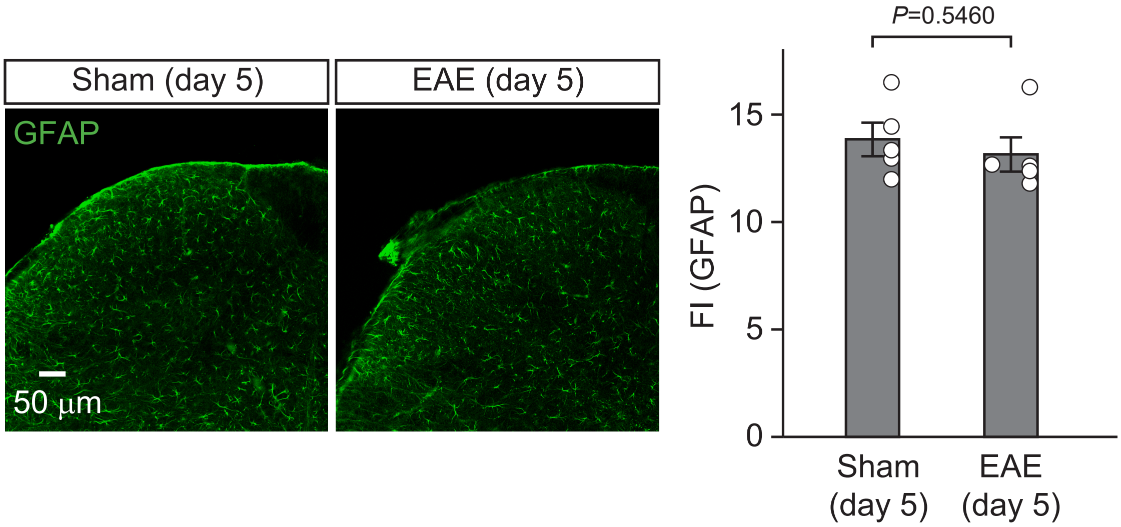

Supplementary Figure 2. Fluorescent images for GFAP in the L5 spinal dorsal horn 5 days after $MOG_{35\text{-}55}$ immunization. Columns represent statistical data of fluorescence intensity (FI) of GFAP in the spinal dorsal horn. n = 5 mice per group, unpaired *t*-test, $P = 0.5460$. All values are mean ± SEM.

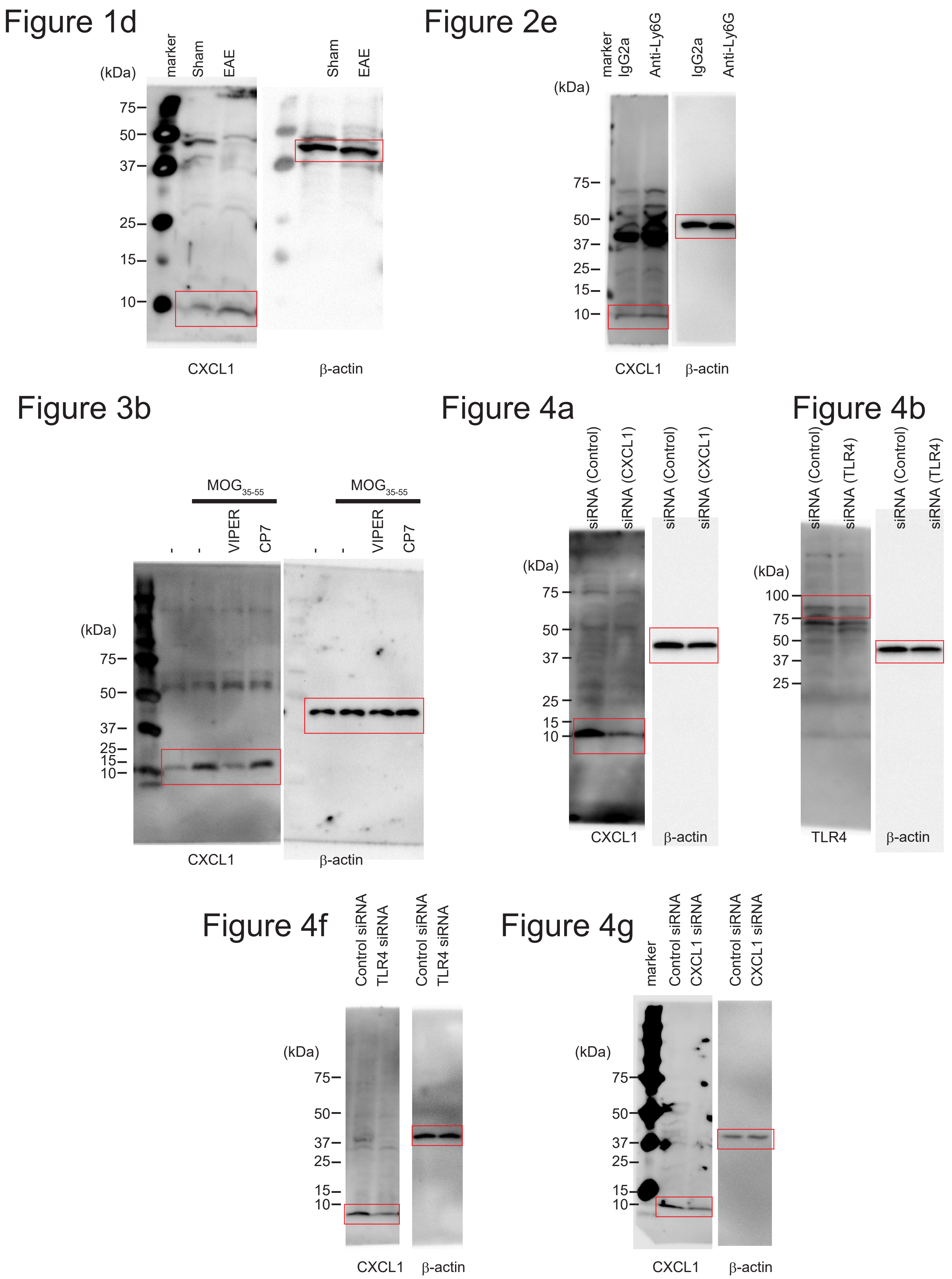

Supplementary Figure 3. Full-length images of blots and gels presented in Figures 1-4. Rectangles indicate the regions used in the figures.
